# Acute Skeletal Muscle Activation Through Physical Exercise and Its Effects on Cognitive Performance and Neurobiological Markers in Adults: A Scoping Review

**DOI:** 10.3390/muscles5020025

**Published:** 2026-03-30

**Authors:** Sabine D. Brookman-May

**Affiliations:** Department of Urology, University of Munich (LMU), 81377 Munich, Germany; sabine.brookman-may@email.de

**Keywords:** acute exercise, cognitive performance, executive function, exercise physiology, brain-derived neurotrophic factor (BDNF), cerebrovascular function, functional near-infrared spectroscopy (fNIRS), high-intensity interval training (HIIT), muscle–brain crosstalk, myokines, skeletal muscle, neuromuscular activation

## Abstract

Physical exercise can influence cognitive performance and neurobiological processes, but evidence spans diverse modalities, intensities, and adult populations. Acute exercise represents a state of transient skeletal muscle activation that induces systemic signaling through metabolic, endocrine, and myokine-mediated pathways, which may contribute to neurocognitive modulation. To map the breadth of acute exercise–cognition research, characterize cognitive and biological outcomes, and identify consistent patterns and gaps. Studies of adults (≥18 years) involving a single exercise session or short microcycle (≤7 days) with pre–post assessment of cognition and/or neurobiological markers across any exercise modality (aerobic, resistance, high-intensity interval training/HIIT, combined, vibration, mind–body) were included. PubMed and CENTRAL were systematically searched, yielding 101 studies. Data were extracted using a structured framework capturing exercise modality, dose, cognitive domains, biomarkers, neuroimaging outcomes, population characteristics, and study design features. Most studies examined young adults (53%) or older adults (32%). Aerobic exercise predominated (62%), followed by resistance (18%) and combined modalities (12%). Moderate-to-vigorous aerobic exercise consistently improved executive function, processing speed, and working memory. Resistance exercise also enhanced executive function in several trials (31 studies). Neurobiological correlates included increases in Brain-Derived Neurotrophic Factor (BDNF), lactate, catecholamines, and prefrontal activation, though variability in sampling limited mechanistic conclusions. Acute exercise is consistently associated with improvements in executive function and processing speed across modalities. Standardized exercise protocols, biomarker timing, and cognitive assessments are needed to strengthen mechanistic synthesis.

## 1. Introduction

Acute bouts of physical exercise can transiently alter multiple neurobiological processes, including neurotrophin release, metabolic signaling, and cerebral oxygenation [1,2,3,4,5]. Early studies demonstrated improvements in cognitive performance following aerobic or near-threshold exercise in both younger and older adults [1,2,3,4]. Subsequent work has shown that high-intensity interval training (HIIT), moderate aerobic exercise, and resistance exercise can all acutely enhance executive function, attention, and memory [5,6,7,8,9,10,11,12].

Across these early investigations, neurobiological responses such as increased Brain-Derived Neurotrophic Factor (BDNF), changes in lactate and cortisol, and altered prefrontal activation were repeatedly observed following acute exercise [6,7,8,9,10,11,12,13,14,15]. Together, these findings provided initial evidence that transient physiological responses may relate to short-term cognitive benefits. From a muscle physiology perspective, acute exercise represents a coordinated activation of skeletal muscle tissue, which functions not only as a contractile system but also as an endocrine organ. Contracting muscle fibers release a range of signaling molecules, including myokines, metabolites such as lactate, and neuroactive factors, which can influence distant organs including the brain. This muscle–brain crosstalk provides a biologically plausible framework linking acute muscular activity to rapid changes in cognitive performance and neurobiological markers.

### 1.1. Rationale

Although prior reviews have explored specific components of this research area—such as BDNF responses to acute exercise, aerobic intensity effects, or short-term executive function changes—these syntheses have typically focused on narrow subsets of the literature [6,7,8,9,10,11,12,13,14,15]. No prior review has mapped the full scope of acute exercise–cognition research across all major exercise modes, biological pathways, and neurophysiological outcomes in adult humans. Given the heterogeneity across exercise modalities, cognitive domains, and outcome measures, a scoping review approach was chosen to systematically map this complex evidence base.

### 1.2. Objectives

**Figure 1:**
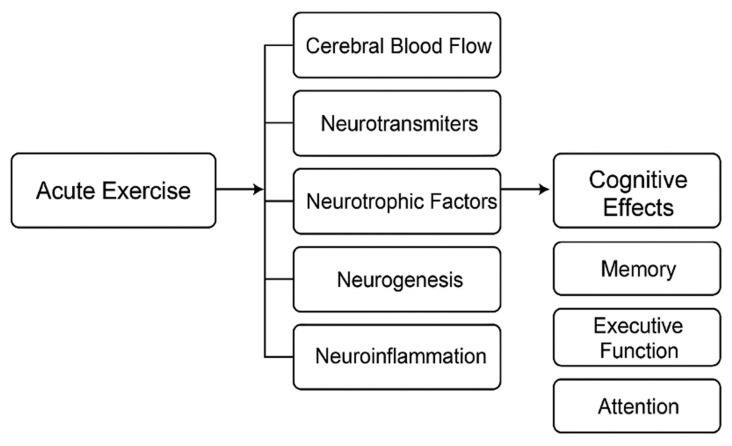
Acute Exercise, neurobiological changes, and cognitive effects.

The present scoping review aggregates 101 human studies on acute exercise and cognition [1,2,3,4,5,6,7,8,9,10,11,12,13,14,15,16,17,18,19,20,21,22,23,24,25,26,27,28,29,30,31,32,33,34,35,36,37,38,39,40,41,42,43,44,45,46,47,48,49,50,51,52,53,54,55,56,57,58,59,60,61,62,63,64,65,66,67,68,69,70,71,72,73,74,75,76,77,78,79,80,81,82,83,84,85,86,87,88,89,90,91,92,93,94,95,96,97,98,99,100,101] with the aims to:

(1) Map the range of exercise modalities and stimuli studied;
(2) Describe the cognitive domains most commonly assessed;
(3) Summarize neurobiological and neurophysiological correlates (e.g., BDNF, lactate, catecholamines, hemodynamics, electroencephalography/EEG, Functional Near-Infrared Spectroscopy/fNIRS, Magnetic Resonance Imaging/MRI);
(4) Highlight consistent findings across exercise types and populations;
(5) Identify research gaps suitable for future mechanistic or translational work (Figure 1).

This scoping review follows a structured search and screening approach (Supplementary Table S1) while maintaining the methodological flexibility appropriate for charting heterogeneous evidence across diverse study designs.

Acute exercise in this review refers to a single exercise session or short-term exposure (≤7 days), distinguishing it from chronic training adaptations. Exercise modalities considered include aerobic, resistance, and interval-based protocols, which may differentially influence cognitive outcomes through distinct physiological mechanisms. Cognitive domains were categorized into executive function (including inhibitory control and cognitive flexibility), working memory, attention/processing speed, and memory/learning, reflecting the most commonly assessed constructs in the literature. This structured framework was applied to ensure consistent mapping of outcomes across heterogeneous study designs and to align the conceptual definitions with the methodological approach of this scoping review.

## 2. Materials and Methods

### 2.1. Protocol and Registration

A scoping review methodology was chosen to accommodate the heterogeneity across study designs, exercise modes, populations, and cognitive outcomes. The review protocol was prospectively registered with the International Prospective Register of Systematic Reviews (PROSPERO; Registration ID: 1229780). Methods followed PRISMA-ScR guidance and were tailored to mechanistic exercise–cognition research [1,2,3,4,5,6,7,8,9,10,11,12,13,14,15,16,17,18,19,20,21,22,23,24,25,26,27,28,29,30,31,32,33,34,35,36,37,38,39,40,41,42,43,44,45,46,47,48,49,50,51,52,53,54,55,56,57,58,59,60,61,62,63,64,65,66,67,68,69,70,71,72,73,74,75,76,77,78,79,80,81,82,83,84,85,86,87,88,89,90,91,92,93,94,95,96,97,98,99,100,101].

### 2.2. Eligibility Criteria

Studies were included if they: 1. Involved human adults (≥18 years); 2. Applied an acute bout of physical exercise (single session or microcycle ≤ 7 days); 3. Measured cognition and/or neurobiological markers pre/post exercise; 4. Used any exercise modality (aerobic, resistance, HIIT, combined, vibration, mind–body); and 5. Reported original human data [1,2,3,4,5,6,7,8,9,10,11,12,13,14,15,16,17,18,19,20,21,22,23,24,25,26,27,28,29,30,31,32,33,34,35,36,37,38,39,40,41,42,43,44,45,46,47,48,49,50,51,52,53,54,55,56,57,58,59,60,61,62,63,64,65,66,67,68,69,70,71,72,73,74,75,76,77,78,79,80,81,82,83,84,85,86,87,88,89,90,91,92,93,94,95,96,97,98,99,100,101]. Animal studies, chronic training interventions, protocol-only reports, and conference abstracts without methods were excluded.

### 2.3. Information Sources

Two electronic databases were searched: PubMed and Cochrane CENTRAL. Searches targeted literature on acute physical exercise, cognitive performance and executive functions, biomarkers such as BDNF and other neurotrophins, and neuroimaging or cortical activation measures (fNIRS, fMRI, EEG, Arterial Spin Labeling-MRI/ASL-MRI) [1,2,3,6,7,8,13,17,18,21,22,23,25,28,29,32,35,39,41,45,47,52,56,57,58,60,63,64,65,69,73,79,80,82,83,86,87,88,89,91,92,93,96,97,98,99]. Full search strings are provided in Supplementary Table S1.

### 2.4. Search

Searches were performed without year restrictions and included all articles available up to the final search date. The final literature search was conducted in November 2026. Titles and abstracts retrieved from PubMed and CENTRAL were exported, deduplicated, and screened. The search strategy was intentionally broad to capture the full spectrum of acute exercise, i.e., cognition paradigms, biomarker assessments, and imaging methods.

### 2.5. Selection of Sources of Evidence

Screening occurred in two stages:

(1) Title/abstract screening using predefined eligibility criteria;
(2) Full-text review for abstracts meeting inclusion criteria or when eligibility was uncertain.

**Figure 2:**
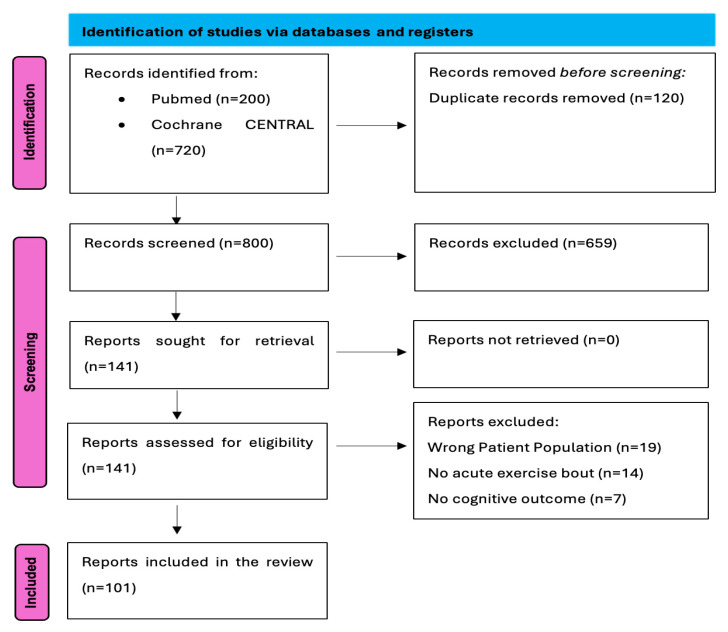
PRISMA Flow Diagram.

A total of 101 studies met all criteria and were included in the review [1,2,3,4,5,6,7,8,9,10,11,12,13,14,15,16,17,18,19,20,21,22,23,24,25,26,27,28,29,30,31,32,33,34,35,36,37,38,39,40,41,42,43,44,45,46,47,48,49,50,51,52,53,54,55,56,57,58,59,60,61,62,63,64,65,66,67,68,69,70,71,72,73,74,75,76,77,78,79,80,81,82,83,84,85,86,87,88,89,90,91,92,93,94,95,96,97,98,99,100,101]. Reasons for exclusion were documented during screening (e.g., chronic interventions, animal models, missing cognitive or biomarker outcomes). Figure 2 displays the PRISMA 2020 flow diagram. Study selection and data charting were performed by a single reviewer using a structured framework. To enhance consistency, eligibility criteria and extraction categories were iteratively refined during the process, in accordance with scoping review methodology.

### 2.6. Data Charting Process

Data were charted using a standardized extraction template. For each included study, we systematically recorded study design and sample characteristics; exercise modality, dose, and intensity; timing of assessments; cognitive domains and specific tasks; biomarker measures (e.g., BDNF, lactate, cortisol, catecholamines); neuroimaging outcomes (fNIRS, fMRI, EEG/Event-Related Potential/ERP, ASL-MRI), and major findings. The charting procedure was iterative: extraction was refined as new patterns emerged, consistent with scoping review methodology. Given the breadth of the included literature, data extraction prioritized key information from abstracts and full texts where available to enable comprehensive evidence mapping.

### 2.7. Data Items

Key data items included:

- Population variables: age group, health status, fitness level.
- Exercise variables: modality (aerobic, resistance, HIIT, combined), session duration, intensity, and environmental conditions (e.g., hypoxia).
- Cognitive variables: executive function, working memory, attention, visuospatial ability, memory/learning.
- Biological variables: neurotrophic factors (BDNF), muscle-derived factors (myokines such as irisin, CTSB), metabolic markers (lactate, glucose), endocrine markers (cortisol, catecholamines), inflammatory markers, myokines.
- Neurophysiological variables: cortical oxygenation, cerebral blood flow, electrophysiological responses, regional activation.
- Outcomes: direction and magnitude of cognitive change, biomarker response, imaging signal change.

### 2.8. Critical Appraisal of Individual Sources of Evidence

Formal risk-of-bias assessment was not performed, consistent with PRISMA-ScR guidance and the exploratory purpose of a scoping review. Instead, methodological characteristics were noted that may influence interpretation of results, including sample size, timing of assessments, biomarker protocols, and cognitive test quality.

### 2.9. Synthesis of Results

A narrative synthesis was conducted, structured around the charted domains. Studies were grouped by exercise modality and intensity; cognitive domain tested; biomarker type; neuroimaging modality; and population characteristics. Patterns were summarized thematically to identify consistent findings, mechanistic trends, and evidence gaps [1,2,3,4,5,6,7,8,9,10,11,12,13,14,15,16,17,18,19,20,21,22,23,24,25,26,27,28,29,30,31,32,33,34,35,36,37,38,39,40,41,42,43,44,45,46,47,48,49,50,51,52,53,54,55,56,57,58,59,60,61,62,63,64,65,66,67,68,69,70,71,72,73,74,75,76,77,78,79,80,81,82,83,84,85,86,87,88,89,90,91,92,93,94,95,96,97,98,99,100,101]. While the primary focus was on acute exercise responses, studies embedded within short-term repeated-session designs were included when acute effects could be clearly delineated.

## 3. Results

### 3.1. Selection of Sources of Evidence

A total of 101 studies met all eligibility criteria and were included in the review [1,2,3,4,5,6,7,8,9,10,11,12,13,14,15,16,17,18,19,20,21,22,23,24,25,26,27,28,29,30,31,32,33,34,35,36,37,38,39,40,41,42,43,44,45,46,47,48,49,50,51,52,53,54,55,56,57,58,59,60,61,62,63,64,65,66,67,68,69,70,71,72,73,74,75,76,77,78,79,80,81,82,83,84,85,86,87,88,89,90,91,92,93,94,95,96,97,98,99,100,101]. These studies were identified through systematic searches of PubMed and CENTRAL, followed by title/abstract screening and full-text review. Reasons for exclusion included chronic training interventions, animal studies, missing cognitive or biomarker outcomes, or insufficient methodological information (Figure 2).

### 3.2. Characteristics of Sources of Evidence

The 101 included studies varied widely in design, population, exercise modality, and measurement methods (Table 1). Most studies involved healthy young adults [3,4,5,6,7,9,10,11,18,19,23,24,25,26,27,30,31,32,33,35,44,47,48,63,64,67,69,70,73,78,81,82,89,90,95,97,99], with additional cohorts of older adults, sedentary individuals, high-level athletes, and groups at elevated metabolic or cognitive risk (e.g., overweight adults, individuals with type 1 diabetes) [1,8,12,13,14,15,16,17,20,21,28,29,34,36,37,38,40,41,42,46,49,51,53,56,59,60,61,62,65,72,74,75,76,77,79,83,84,85,86,88,91,93,100,101].

Exercise modalities included aerobic exercise, HIIT, resistance exercise, combined aerobic–resistance protocols, open-skill tasks, and a range of less common modes (eccentric cycling, aquatic treadmill, vibration, dual-task Blood Flow Restriction/BFR, orienteering) [2,5,7,8,9,12,20,24,27,31,34,40,41,46,47,48,49,50,52,53,60,66,70,71,72,77,85,88,90,94,100]. Dual-Task paradigms represent more complex or multimodal interventions that may introduce additional cognitive load and should therefore be interpreted with caution.

Cognitive assessments spanned executive function, working memory, attention, task switching, visuospatial processing, motor learning, and implicit/explicit memory [6,13,15,19,24,28,31,34,35,41,45,46,47,48,51,53,60,63,68,70,72,81,82,84,88,90,91,92,95,96,97,98,99,101]. The strength of evidence varied across cognitive domains, with the most consistent findings observed for executive function and inhibitory control, while other domains such as memory and global cognition showed more heterogeneous results.

Neurobiological outcomes included BDNF [3,5,7,9,10,11,12,13,14,15,17,18,28,32,38,39,40,42,46,48,50,56,62,65,72,76,77,79,83,85,88,91], endocrine markers [3,5,7,9,10,11,12,13,14,15,16,17,18,20,28,32,33,34,38,39,40,41,42,44,46,47,48,49,50,51,62,64,65,72,76,77,79,83,85,88,91,95], inflammatory mediators [19,34,41,44,49,51,95], and brain-physiological effects measured by EEG/ERP, fNIRS, ASL or Blood Oxygenation Level-Dependent (BOLD), fMRI, and cerebrovascular hemodynamics (middle cerebral artery velocity, cerebral blood flow/CBF redistribution, prefrontal oxygenation) [2,4,21,22,23,24,25,26,27,31,33,36,37,43,52,57,58,59,60,61,66,67,68,70,71,74,75,81,87,90,94,96,98].

### 3.3. Critical Appraisal Within Sources of Evidence

Consistent with scoping review methodology, a formal risk-of-bias appraisal was not performed. However, methodological variability across studies was noted in sample sizes, control conditions, exercise dosing, cognitive task timing, biomarker protocols (serum vs. plasma BDNF), and neuroimaging acquisition methods. These factors likely influenced heterogeneity in observed outcomes.

### 3.4. Results of Individual Sources of Evidence

To preserve scientific clarity, the following subsections summarize findings by outcome domain (Table 2).

#### 3.4.1. Effects of Acute Exercise on Executive Function

Across approximately 60 of the included studies, acute exercise elicited measurable improvements in executive function, particularly for tasks involving inhibition, task switching, updating, and dual-task processing [13,15,19,24,28,31,34,35,41,45,46,51,53,60,63,68,70,72,81,82,84,90,91,92,95,96,97,98,99,101]. Improvements were observed within minutes after exercise cessation and typically persisted for up to 30–60 min, although some studies reported effects lasting longer [13,19,24,34,60,63,72,84,91] (Table 3).

Moderate-intensity aerobic exercise produced reliable improvements in Stroop performance, trail-making time, response inhibition, and task-switching speed [13,15,19,34,45,54,60,63,72,91]. Several studies using cycling or treadmill walking at 60–70% of maximal heart rate demonstrated enhanced Stroop performance with concomitant increases in prefrontal oxygenation [14,19,24,32,38,54,63,72,82].

HIIT frequently produced equal or superior improvements, particularly in tasks requiring rapid inhibitory control or working memory updating [2,7,8,17,27,41,60,65,67,70,84,94,100]. Across the included HIIT studies (n = 14), HIIT produced larger and sometimes more persistent effects than moderate-intensity exercise, with executive benefits often extending throughout the recovery period (20–30 min post-exercise) [2,8,60,67,84]. Higher-intensity exercise also elicited stronger neuroelectric signatures (e.g., increased P3 amplitude and enhanced Contingent Negative Variation/CNV) in several ERP-based trials [13,27,41].

However, HIIT responses were not universal. A minority of studies reported no superiority of HIIT over traditional moderate-intensity exercise or even null cognitive effects, suggesting that individual fitness levels and protocol design strongly shape outcomes [59,73,75].

*Effects of resistance and multimodal exercise*

Evidence from resistance training (RT) demonstrates that both low-intensity slow-movement resistance exercise and high-intensity resistance exercise can improve inhibitory control and executive function [5,9,20,27,49,55,70,85,88,100]. Combined resistance + power training or concurrent aerobic–resistance protocols reliably improved global cognition, short-term memory, and dual-task performance in older women and middle-aged adults [20,27,49,50,52,70,85].

*Open- versus closed-skill exercise*

Three studies evaluating open-skill activities (e.g., orienteering, racquet-type tasks) showed larger improvements in inhibitory control compared to matched-intensity closed-skill exercise, alongside unique neural efficiency effects with reduced prefrontal activation despite improved performance [31,66,77]. These paradigms, which require constant adaptation and spatial navigation, appear to place additional demands on attentional and visuospatial systems.

#### 3.4.2. Effects on Working Memory and Attention

Overall, more than 30 studies assessed working memory, using n-back tasks, digit span, spatial working memory tests, or memory-recognition paradigms [1,6,13,18,22,24,28,29,41,45,46,63,68,70,81,82,86,93,96,97,98,99] (Table 4). Acute moderate exercise reliably reduced reaction times in both low- and high-load conditions and, in high-fit individuals, increased P3 amplitude and frontal CNV, indicating enhanced neural resource allocation [13,22,24,28,45,63,68,94].

Fitness level modulated outcomes: higher-fit participants showed larger improvements, particularly under high working-memory loads [22,28,63,68,73,94]. Inter-individual differences were notable, with responders exhibiting greater right ventrolateral prefrontal cortex activation during exercise, while non-responders showed minimal fNIRS activation changes and smaller cognitive gains [29,68,72,82,99].

Evidence for sustained attention and vigilance was mixed. Several studies reported improvements in attentional accuracy after moderate or high-intensity sessions [13,24,31,41,51,60,70,94], but two studies using prolonged vigilance tasks found that passive or light exercise did not mitigate mental fatigue [31,78], suggesting that long-duration vigilance paradigms may be less sensitive to acute exercise benefits.

Overall 15 studies investigated episodic memory, motor learning, or implicit/explicit sequence learning [1,6,21,28,35,39,48,53,81,86,93,95,96,97,98]. Several trials demonstrated that acute exercise enhances recognition memory, associative learning, vocabulary learning, and motor sequence encoding when exercise is performed immediately before encoding or training [1,6,21,28,35,81,86,93,96]. Exercise before encoding improved parietal activation, accelerated learning rates, and strengthened retention 24 h later [6,21,39,81,93,96].

In older adults, increases in BDNF after exercise correlated with better post-exercise learning in some cohorts, supporting a mechanistic link between neurotrophic signaling and training gains [18,23,63,80]. Conversely, exhaustive exercise or very high-intensity protocols sometimes reduced motor skill learning performance, potentially due to transient fatigue, altered Supplementary Motor Area (SMA) excitability, or elevated lactate interfering with motor circuits [5,48,92,97,100].

#### 3.4.3. Neurovascular and Hemodynamic Responses

*Prefrontal oxygenation (fNIRS)*

Across nearly 25 studies using fNIRS, mild-to-moderate intensity exercise consistently increased oxyhemoglobin in dorsolateral and ventrolateral prefrontal regions during executive task performance [11,14,19,24,29,32,38,41,44,46,47,54,60,63,66,68,70,72,77,82,83,91,99,101]. These changes frequently aligned with improved accuracy and reduced reaction times in Stroop, Flanker, and task-switching paradigms [13,19,24,41,45,46,60,63,72,82,83,91]. Prefrontal oxygenation on fNIRS, brain hemodynamics and imaging outcomes across studies are summarized in Table 5.

Vigorous or exhaustive exercise, in contrast, sometimes caused a decrease in prefrontal oxyhemoglobin, consistent with transient hypofrontality under severe metabolic demand [5,31,40,48,60,92,100]. Enhanced oxygenation during or immediately after exercise correlated with improved cognitive performance in many, but not all, studies [11,14,19,24,29,44,54,63,72,82,99].

*Cerebral blood flow (CBF) and cerebrovascular responses*

Studies employing ASL-MRI, Transcranial Doppler (TCD), or phase-contrast MRI reported heterogeneous but interpretable patterns [33,38,39,42,43,51,58,61,63,71,90,92]. Global CBF often decreased immediately after moderate-to-vigorous exercise, while regional CBF responses differed by area, with motor cortex blood flow increasing during exercise and hippocampal or frontal regions showing decreases or complex redistribution [33,39,58,63,71,90,92]. The uncoupling between global CBF and cognitive performance suggests that regional cerebrovascular redistribution, rather than overall flow, may support improved cognitive performance despite global reductions [33,39,42,58,63,71,90,92].

*Impact of hypoxia*

Four studies evaluated hypoxic conditions. Exercise in moderate normobaric hypoxia impaired reaction times but preserved accuracy in some tests, even when BDNF increased [43,56,57,61]. Combined hypoxia and cognitive challenge reduced prefrontal oxygenation further, suggesting a competitive resource limitation for cortical control processes [43,56,57,61].

#### 3.4.4. Brain-Derived Neurotrophic Factor (BDNF)

Across all BDNF-focused studies (n = 40), roughly 70% reported increases in circulating BDNF after acute exercise (Table 6; [1,2,3,6,7,8,10,13,17,18,21,22,23,25,28,29,41,45,52,56,57,58,60,63,64,65,69,73,79,80,86,87,88,89,93,96,97,98]). Increases were observed across modalities—continuous aerobic exercise, HIIT, resistance exercise, whole-body vibration, and multimodal protocols [1,2,3,5,6,7,8,9,13,17,18,20,21,22,23,27,41,45,49,52,59,60,64,73,79,80,85,86,87,88,89,93,96,97,98]. The magnitude of response varied widely and was influenced by fitness level, exercise intensity, sex, timing, and whether serum or plasma was sampled [1,2,3,10,17,18,21,22,23,25,28,41,52,64,69,73,79,80,86,87,88,89].

Most studies did not find a tight correlation between BDNF changes and acute cognitive changes [3,10,21,22,23,28,41,45,52,59,69,73,79,80,87,88,89]. Exceptions included work in older adults where larger BDNF increases predicted greater post-exercise learning or working-memory gains [18,23,63,80]. In several high-intensity protocols, lactate levels appeared to mediate BDNF responses, although not necessarily behavioral outcomes [5,7,8,9,41,60,73,79,87,100].

Null BDNF findings were also common. A smaller subset of studies reported no increase in BDNF after exercise—for example, cycling near heavy traffic, periods of detraining, or HIIT combined with β-alanine [37,59,69,75,76,89]. These trials highlight substantial interindividual variability and the modifying roles of environmental exposures and chronic activity levels [22,28,37,69,73,75,76].

#### 3.4.5. Other Biological Markers

Beyond BDNF, studies assessed cortisol, catecholamines, DHEA, inflammatory markers, myokines, and endocrine responses [5,7,8,9,10,21,25,28,30,39,41,50,55,59,70,73,79,85,90,93,100]. HIIT and exhaustive exercise tended to provoke larger cortisol and catecholamine surges than moderate continuous training [7,8,9,25,41,59,70,73,79,100]. Acute exercise generally reduced inflammatory signaling or shifted apoptotic mediators in more favorable directions, especially in older adults or aging populations engaging in functional or multicomponent programs [30,33,50,52,70,85]. Several studies highlighted exercise-induced changes in myokines (e.g., irisin, CTSB) and IGF-1 as potential contributors to neuroplasticity, though associations with acute cognitive changes were still exploratory [5,7,8,9,25,41,59,70,73,79,87,100]. These findings further support the concept that skeletal muscle acts as a central mediator of systemic exercise responses, with contraction-induced signaling contributing to downstream neurobiological and cognitive effects.

#### 3.4.6. Influence of Age, Fitness, and Baseline Capacity

Older adults consistently benefited from acute exercise but often showed smaller or more variable effect sizes compared with younger individuals [11,15,18,21,22,23,33,35,42,44,49,50,52,63,69,72,80,85,90,98]. Robust Stroop and working-memory improvements were seen after moderate-intensity cycling or walking, frequently accompanied by increased prefrontal activation or CBF [11,15,19,23,33,42,44,63,72]. In older cohorts, associations between exercise-induced BDNF elevations and improved working memory or learning appeared particularly prominent [18,23,63,80].

Cardiorespiratory fitness consistently modulated cognitive and biomarker responses. Higher fitness predicted larger executive-function improvements, stronger ERP signatures, and greater BDNF responsiveness in several cohorts [21,22,28,45,63,68,73,80,94]. Conversely, lower-fit individuals benefitted from acute exercise but showed more modest neural signatures and smaller biomarker shifts [22,28,37,69,73,75].

Clinical and at-risk populations—such as individuals with type 1 diabetes or those at elevated risk for mild cognitive impairment—appeared to derive benefit from acute interventions, though mechanisms may be altered by baseline inflammation or vascular stiffness [33,37,69,74,85]. Evidence from these groups, while limited, suggests that acute exercise remains a promising adjunct even when chronic disease is present [33,69,74,85,90].

#### 3.4.7. Task- and Modality-Specific Effects

Task- and modality-specific patterns emerged across the dataset. Open-skill exercise and navigation-based paradigms (e.g., orienteering) produced some of the strongest improvements in inhibitory control and spatial cognition, often with reduced prefrontal activation consistent with enhanced neural efficiency [31,66,72,77]. Concurrent or multicomponent exercise programs yielded cognitive benefits comparable to aerobic training, with lactate and myokine responses implicated as potential mediators [7,8,9,20,27,41,49,50,52,70,79,87,100]. Aquatic treadmill exercise improved Digit Symbol performance and cerebral blood flow in older adults [44]. Eccentric cycling and high-intensity functional training were associated with higher mental demand and distinct frontoparietal activation patterns, despite equivalent or lower external workloads [31,40,70,92,100]. Intermittent squat or walking breaks during prolonged sitting preserved executive function and cerebrovascular responsiveness compared with uninterrupted sitting [51].

Taken together, the 101 included studies demonstrate that acute exercise reliably improves executive function, working memory, and inhibitory control across intensity levels, modalities, and populations (Table 7, [1,2,3,4,5,6,7,8,9,10,11,12,13,14,15,16,17,18,19,20,21,22,23,24,25,26,27,28,29,30,31,32,33,34,35,36,37,38,39,40,41,42,43,44,45,46,47,48,49,50,51,52,53,54,55,56,57,58,59,60,61,62,63,64,65,66,67,68,69,70,71,72,73,74,75,76,77,78,79,80,81,82,83,84,85,86,87,88,89,90,91,92,93,94,95,96,97,98,99,100,101]).

HIIT often elicits the strongest and most durable cognitive benefits, but moderate-intensity exercise remains highly effective and more universally tolerated [2,7,8,13,15,19,24,34,41,45,46,59,60,63,72,73,84,91,94]. Prefrontal oxygenation consistently increases during mild or moderate exercise but may decrease under high physiological load [11,14,19,24,29,32,38,40,46,54,60,63,70,72,82,92,99]. BDNF increases in most trials but does not correlate tightly with cognitive improvements, underscoring a multi-factorial mechanism [1,2,3,6,7,8,17,18,21,22,23,25,28,41,45,52,56,57,58,64,69,73,79,80,86,87,88,89,93,96,97,98]. Effects vary by age, fitness, exercise modality, and task type, and acute exercise induces complex neurovascular responses, including regional CBF redistribution and transient hypofrontality at high loads [33,38,39,42,43,51,58,61,63,71,90,92]. Open-skill and cognitively demanding exercises may further enhance neural efficiency, improving performance with reduced cortical activation [31,66,72,77,99,101].

## 4. Discussion

**Figure 3:**
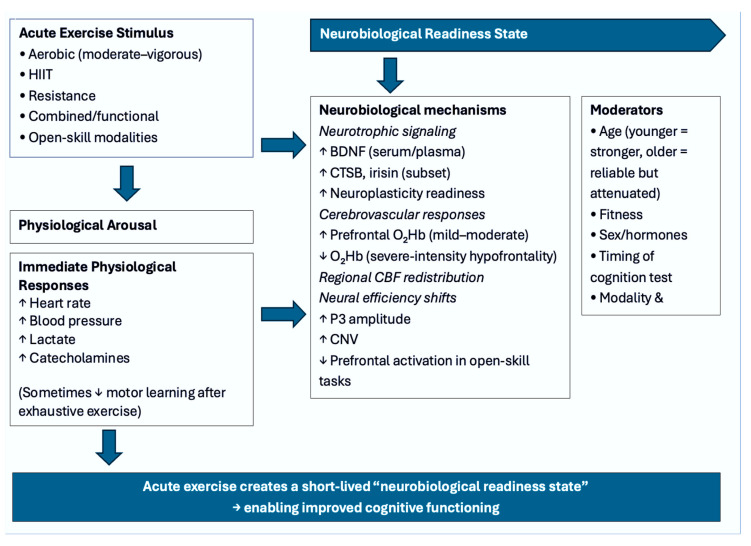
Model of Acute Exercise Stimulus and Neurobiological Readiness.

The principal aim of this scoping review was to map and synthesize the acute cognitive, neurotrophic, vascular, and mechanistic effects of single-session exercise across 101 empirical studies [1,2,3,4,5,6,7,8,9,10,11,12,13,14,15,16,17,18,19,20,21,22,23,24,25,26,27,28,29,30,31,32,33,34,35,36,37,38,39,40,41,42,43,44,45,46,47,48,49,50,51,52,53,54,55,56,57,58,59,60,61,62,63,64,65,66,67,68,69,70,71,72,73,74,75,76,77,78,79,80,81,82,83,84,85,86,87,88,89,90,91,92,93,94,95,96,97,98,99,100,101]. Despite heterogeneity in populations, exercise modalities, intensities, testing batteries, and biomarkers assessed, several robust themes emerged (Figure 3). Collectively, the evidence indicates that acute exercise—whether aerobic, resistance-based, multimodal, or delivered as HIIT—produces consistent small-to-moderate improvements in executive function, frequently accompanied by transient neurotrophic and hemodynamic changes [5,6,7,8,9,13,15,17,18,19,20,21,23,24,25,27,28,31,34,39,40,41,44,45,46,47,49,50,52,60,63,68,70,72,79,80,81,82,84,90,91,92,93,96,97,98,99,100,101]. Importantly, these effects appear independent of age, sex, or baseline fitness, but their magnitude is modulated by exercise intensity, individual cardiorespiratory fitness, task timing relative to exercise, and neurobiological responsiveness [15,18,21,22,23,28,33,37,42,44,63,68,69,72,73,80,85,94].

(A) Skeletal Muscle as a Central Mediator of Exercise–Brain Interaction

Skeletal muscle plays a central role in mediating the systemic effects of acute exercise and can be considered a primary driver of exercise–brain interactions. Beyond its mechanical function, contracting muscle acts as an endocrine organ, releasing bioactive molecules, including myokines, metabolites such as lactate, and neuroactive factors, that influence central nervous system function. These signals modulate neurotrophic pathways, cerebral blood flow, and neuronal activity, providing a mechanistic link between peripheral muscle activation and cognitive enhancement. This framework positions skeletal muscle as a key mediator of exercise-induced neurobiological adaptations and a potential therapeutic target for optimizing brain health.

(B) Acute Exercise Produces Rapid and Reliable Cognitive Enhancement

Across studies, acute exercise most consistently improved executive function—including inhibitory control, task-switching, cognitive flexibility, and working memory [13,15,19,24,28,31,34,41,45,46,51,53,60,63,68,70,72,81,82,84,90,91,92,95,96,97,98,99,101]. Beneficial effects were detected after moderate-intensity continuous cycling, brisk running, resistance exercise, HIIT, whole-body vibration–assisted training, eccentric exercise, aquatic treadmill walking, and brief sprint sessions [2,5,6,7,8,9,13,15,19,20,24,27,31,34,41,44,45,46,49,50,52,54,60,63,66,70,72,77,85,88,90,94,100]. Classical demonstrations include improved Stroop or Flanker performance and task-switching after moderate cycling or walking near the anaerobic threshold, enhanced executive function after HIIT protocols, and improved dual-task walking performance after acute exercise in older adults [13,15,19,24,34,45,46,60,63,72,91].

*Intensity matters—but not in the way typically assumed*

Several studies reported an inverted-U relationship, with moderate-to-near-threshold intensities producing optimal cognitive outcomes [13,15,19,34,45,54,60,63,72,91]. Exercising at workloads close to the anaerobic threshold in older women enhanced multiple executive domains, whereas very mild or very intense interventions often produced attenuated or selective benefits [15,31,44,48,60,92].

At the same time, HIIT studies contradict a simple intensity narrative. Multiple investigations showed HIIT to produce equal or superior cognitive gains compared with moderate steady-state exercise, particularly on tasks with high inhibitory or working-memory demands [2,7,8,17,27,41,60,65,67,70,84,94,100]. However, the window of improvement may be shorter and may dissipate more rapidly during recovery, with repeated HIIT bouts shortening the duration of post-exercise facilitation in some trials [60,84,92].

*Exercise type is less important than perceived effort*

When subjective intensity was matched, different modalities (aerobic, resistance, and balance-focused multicomponent exercise) produced comparable improvements in inhibitory control and global cognition [20,27,34,49,50,52,70,85,88]. This suggests that cognitive gains are largely driven by global physiological arousal and neuromodulatory engagement rather than strictly modality-specific factors.

(C) Neurobiological Mechanisms: BDNF as the Central but Not Exclusive Mediator

BDNF emerged as the most frequently investigated mechanistic biomarker [1,2,3,6,7,8,10,13,17,18,21,22,23,25,28,41,45,52,56,57,58,60,63,64,65,69,73,79,80,86,87,88,89,93,96,97,98]. The majority of studies demonstrated acute BDNF increases following aerobic or high-intensity exercise, and in several trials these surges were temporally linked to faster task-switching, improved working memory, or better learning performance [1,3,6,7,8,17,18,21,23,28,41,45,52,63,73,79,80,86,87,93,96,97,98].

Remarkably, the relationship between BDNF elevation and learning was time-dependent: when cognitive training immediately followed physical exercise, greater BDNF rises predicted superior training gains in older adults [18,23,63,80], but not when the temporal order was reversed.

*Not all BDNF responses are equal*

Studies measuring both serum and plasma revealed divergent kinetics across compartments, reflecting platelet release dynamics rather than purely central production [1,2,3,10,21,22,25,52,64,69,80,86,87,88,89]. Still, the acute changes were often associated with cognitive outcomes, consistent with BDNF acting as a biomarker of neuroplastic readiness rather than a single causal mediator.

Several studies demonstrated no BDNF increase in response to acute exercise, and high interindividual variation was common [37,59,69,75,76,89]. Environmental elements (such as traffic pollution during outdoor cycling), cardiorespiratory fitness, inflammatory milieu, and metabolic status emerged as modulators of the BDNF response [22,28,30,37,69,73,75,76].

(D) Cerebral Blood Flow, Oxygenation, and Hemodynamics: A Rapid but Transient Pathway

Brain perfusion, cortical oxygenation, and cerebrovascular responsiveness were frequently assessed using fNIRS, ASL-MRI, TCD, and other near-infrared methods [11,14,19,24,29,32,33,38,39,42,43,44,51,54,58,61,63,71,72,90,92,99].

*Prefrontal oxygenation increases during and after exercise*

Multiple fNIRS studies demonstrated increases in prefrontal oxyhemoglobin during executive tasks performed after moderate exercise, often aligning with performance gains [11,14,19,24,29,32,38,41,44,46,54,63,66,68,70,72,82,83,91,99,101]. These patterns were observed during moderate cycling, brisk walking, running, HIIT, and combined paradigms with vibration or blood-flow restriction [7,11,14,19,20,24,32,41,44,46,47,54,60,63,66,70,72,83,91,99,100].

*The relationship between oxygenation and cognition is not linear*

Some studies reported reduced cortical activation despite improved performance, suggesting increased neural efficiency rather than increased resource allocation [29,31,66,68,70,72,77,99]. Others showed no direct association between global CBF changes and cognitive gains, particularly when CO_2_ was experimentally manipulated [16,33,38,39,42,58,63,71,90]. Severe-intensity or prolonged exercise often produced temporary declines in cognitive performance during exercise, likely reflecting competition between motor and cognitive demands and transient hypofrontality [5,31,40,48,60,71,90,92,100].

*Hemodynamic responses differ across modalities and environments*

Comparisons between upright and recumbent cycling, aquatic treadmill walking, and eccentric cycling illustrated how posture, hydrostatic pressure, and contraction type influence prefrontal oxygenation and cerebrovascular responses [31,40,44,54,71,90]. Hypoxic trials demonstrated that moderate normobaric hypoxia attenuated cognitive performance and altered hemodynamics even when BDNF rose, underscoring that vascular constraints can override neurotrophic facilitation [43,56,57,61].

(E) Lactate, Catecholamines, Blood Pressure, and Autonomic Nervous System: Fast-Acting Physiological Modulators

Acute lactate responses are increasingly recognized as contributors to exercise-induced cognitive changes. Several studies showed that higher lactate concentrations correlated with improved executive function or working memory, particularly in HIIT, strength-based, or concurrent exercise protocols [5,7,8,9,41,59,60,73,79,87,100]. Norepinephrine, IGF-1, and endocrine markers also rose following certain resistance or vibration-assisted protocols and were linked to working-memory accuracy in some cohorts [5,7,9,20,25,41,55,73,79,85,100]. A recent crossover trial suggested that systolic blood pressure may partially mediate post-resistance improvements in executive function, emphasizing the role of cardiovascular arousal in triggering acute neurocognitive benefits [27].

An additional integrative perspective to these findings is the role of autonomic nervous system (ANS) regulation as a potential upstream mediator of exercise-induced neurobiological and cognitive responses. Acute exercise, particularly HIIT, is characterized by rapid sympathetic activation, followed by a phase of parasympathetic reactivation during recovery. This dynamic interplay may shape neurocognitive outcomes through several pathways, including transient increases in catecholamines, modulation of cerebral perfusion, and metabolic signaling via lactate.

Emerging evidence suggests that the magnitude and temporal pattern of autonomic activation may influence neurotrophic responses such as BDNF release, as well as executive-function performance, particularly during the early recovery phase. High-intensity exercise appears to induce stronger sympathetic stimulation and greater physiological arousal, whereas moderate continuous exercise may promote a more balanced autonomic profile with sustained parasympathetic engagement. These modality-specific autonomic patterns may partly explain differences in cognitive outcomes observed across exercise types.

However, direct mechanistic links between ANS activity, neurotrophic signaling, and cognitive performance remain insufficiently characterized. Most studies assess these components in isolation, and few integrate autonomic measures such as heart rate variability with neurobiological and cognitive endpoints. Future studies combining autonomic, metabolic, and neurotrophic measurements will be critical to better understand the role of ANS regulation as a unifying mechanism in exercise–cognition interactions.

(F) Age, Fitness, and Cognitive Status as Moderators

*Older adults benefit consistently, but mechanisms differ*

Older participants showed robust Stroop and working-memory improvements after moderate exercise and multicomponent programs, often accompanied by increased prefrontal activation and BDNF responses [11,15,18,21,22,23,33,35,42,44,49,50,52,63,69,72,80,85,90]. However, several studies noted slower return-to-baseline hemodynamic responses and greater interindividual variability in biomarker changes in older cohorts [11,18,23,42,44,63,72,80].

*Higher fitness predicts larger cognitive gains*

Multiple studies reported stronger executive-function improvements, higher baseline cognitive scores, and more pronounced ERP or BDNF responses in fitter individuals [21,22,28,45,63,68,73,80,94]. Fitness also modulated responsiveness to HIIT and to combined physical–cognitive interventions, suggesting that individual conditioning status should be considered when prescribing exercise as a cognitive enhancer [22,28,45,63,68,73,80,94].

*Clinical populations show attenuated or delayed responses*

Few included studies specifically targeted individuals with disease or elevated dementia risk, but available data from type 1 diabetes, overweight or metabolically impaired adults, and MCI-risk populations suggest that acute interventions remain beneficial, though mechanisms may be altered by baseline inflammation, vascular stiffness, or neurodegeneration [33,37,69,73,74,85,90]. These findings justify further mechanistic and translational research in clinical cohorts.

(G) Methodological Considerations and Diversity of Approaches

This evidence base is large but methodologically heterogeneous. Most studies used small samples (often N = 15–40) and rarely included preregistration, standardized intensity prescriptions, or harmonized cognitive test batteries [1,2,3,5,6,7,8,9,13,15,17,18,19,20,21,23,24,25,26,27,28,29,31,32,33,34,35,39,40,41,43,44,45,46,47,49,50,51,52,53,56,57,58,59,60,63,64,65,68,70,72,73,75,79,80,81,84,90,91,92,93,95,96,97,98,99,100]. Biomarker assays varied in timing, fasting status, and compartment (serum vs. plasma), complicating direct comparisons [1,2,3,10,21,22,23,25,28,52,64,69,73,79,80,86,87,88,89,93]. In addition, mechanistic pathways should be interpreted with caution, as correlations do not establish causal relationships. Nevertheless, several consistent methodological themes emerged: cognitive assessments conducted immediately after exercise were the most sensitive; executive-function tasks (Stroop, Flanker, TMT-B, n-back) showed the largest and most reliable effects; fNIRS has become the dominant tool for mapping prefrontal responses; and HIIT plus moderate-intensity continuous exercise are the most commonly studied modalities [11,13,14,19,24,29,31,32,38,41,45,46,47,54,60,63,66,68,70,72,82,83,91,94,99,101].

**Figure 4:**
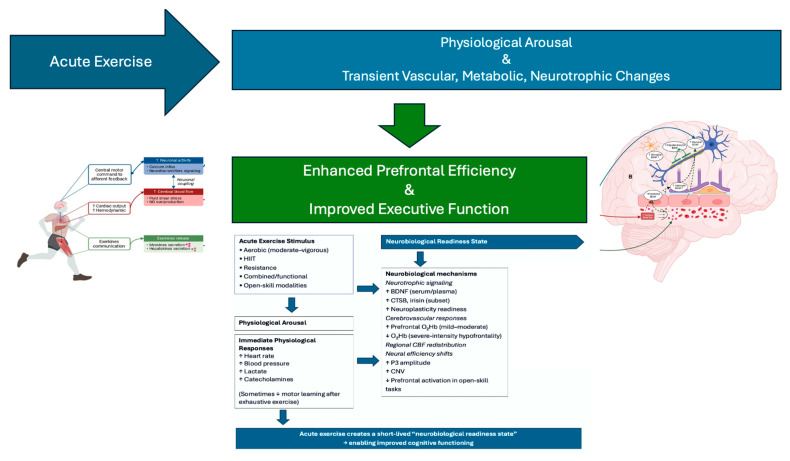
Acute Exercise → Mechanistic Readiness Model.

A unified mechanistic model is therefore emerging (Figure 4):

Acute exercise → physiological arousal → transient vascular, metabolic, and neurotrophic changes → enhanced prefrontal efficiency → improved executive function [1,2,3,5,6,7,8,9,13,14,15,17,18,19,20,21,22,23,25,29,31,32,33,34,35,38,39,40,41,44,45,46,47,52,56,57,58,60,63,64,65,68,70,72,73,79,80,82,83,86,87,88,89,90,91,92,93,96,97,98,99].

(H) Integrative Interpretation and Implications for Practice

Taken together, the evidence suggests several clear implications. Acute exercise is a reliable cognitive enhancer across age groups, with even 10–20 min of moderate or interval-based exercise able to improve key executive processes relevant for academic performance, work productivity, and fall prevention [13,15,19,24,34,41,45,46,60,63,68,70,72,81,82,84,90,91,92,95,96,97,98,99,101]. Exercise intensity should be individualized: moderate-to-ventilatory-threshold workloads and well-designed HIIT protocols appear optimal, but low-volume or mild-intensity interventions can be effective in older or clinical populations, especially when they increase prefrontal efficiency [11,15,19,24,34,44,54,60,63,72,91]. Acute exercise may “prime the brain” for learning or rehabilitation—an idea supported by studies linking post-exercise BDNF changes and vascular responses to improved memory and training outcomes [6,18,21,23,33,39,63,80,81,86,93,96,97,98].

Mechanistic heterogeneity reflects real biological diversity: BDNF, lactate, blood pressure, norepinephrine, inflammatory mediators, and cerebrovascular dynamics all contribute in different populations and contexts [1,2,3,5,6,7,8,9,10,17,18,21,22,23,25,28,30,39,41,50,52,56,57,58,59,69,70,73,79,80,85,86,87,88,89,93,96,97,98,99,100]. This underscores the need for stratified, precision-exercise approaches rather than one-size-fits-all prescriptions.

(I) Future Directions

This scoping review highlights several priorities for future research:

- Standardization: development of common cognitive test batteries, biomarker panels, and sampling time points for acute exercise trials [1,2,3,6,7,8,13,18,21,22,23,25,28,45,52,63,69,73,79,80,86,87,88,89,93,96,97,98,99];
- Multimodal imaging: integration of fNIRS with MRI, EEG/ERP, and Doppler-based methods to link neurovascular, neuroelectric, and neurotrophic changes in the same individuals [11,14,19,24,29,32,33,38,39,41,58,63,71,82,83,90,92,99];
- Larger, preregistered trials are required which are adequately powered to test mechanistic hypotheses and moderators like sex, fitness, and genetics [18,21,22,23,28,33,42,44,63,68,69,73,80,85,94];
- Stratification: consideration of age, hormonal status, baseline cognitive capacity, and genetic markers such as BDNF Val66Met or APOE4;
- Combined interventions: exploration of synergistic protocols combining exercise with cognitive training, nutritional strategies, neuromodulation, or pharmacologic agents [6,18,21,23,28,33,39,41,52,63,73,79,80,86,93,96,97,98].

Limitations

This scoping review has several limitations inherent to the breadth and heterogeneity of the included evidence. First, and in line with the scoping objective of mapping a broad and heterogeneous evidence base, data extraction focused on key information from abstracts and full texts where available, which restricted the ability to examine methodological nuance, timing details, or statistical rigor. Second, substantial variability in exercise protocols (intensity definitions, duration, modality), cognitive task selection, and biomarker sampling methods (serum vs. plasma BDNF, timing of blood draws, preprocessing differences) limited direct comparability across studies. Third, neuroimaging and cerebrovascular measures differed widely in instrumentation and analytic pipelines, making mechanistic interpretation challenging. Fourth, because scoping reviews do not perform formal risk-of-bias assessments, the internal validity of individual studies cannot be assured, and small sample sizes—common across acute-exercise trials—may inflate effect estimates or produce inconsistent findings. Fifth, publication bias and selective reporting likely influenced the available evidence, particularly for positive cognitive outcomes and biomarker changes. Finally, because most studies were conducted in young, healthy adults, generalizability to older adults, clinical populations, or individuals with cognitive impairment remains limited. These factors underscore the need for standardized experimental frameworks and harmonized outcome measures in future mechanistic trials.

## 5. Conclusions

This scoping review provides a comprehensive mapping of the acute cognitive, neurotrophic, and cerebrovascular effects of single-session exercise across healthy and aging adults [1,2,3,4,5,6,7,8,9,10,11,12,13,14,15,16,17,18,19,20,21,22,23,24,25,26,27,28,29,30,31,32,33,34,35,36,37,38,39,40,41,42,43,44,45,46,47,48,49,50,51,52,53,54,55,56,57,58,59,60,61,62,63,64,65,66,67,68,69,70,71,72,73,74,75,76,77,78,79,80,81,82,83,84,85,86,87,88,89,90,91,92,93,94,95,96,97,98,99,100,101]. Despite substantial heterogeneity in study designs, cognitive assessments, biomarkers, and exercise modalities, several consistent findings emerged.

First, acute exercise reliably enhances executive function—particularly inhibitory control, cognitive flexibility, and working memory—across aerobic, resistance, interval-based, balance, and multimodal sessions, as long as sufficient physiological arousal is achieved [5,6,7,8,9,13,15,19,20,24,27,31,34,41,44,45,46,49,50,52,54,60,63,66,70,72,77,81,82,84,88,90,91,92,94,95,96,97,98,99,100,101].

Second, exercise intensity emerges as a key modulator, with moderate-to-ventilatory-threshold workloads and HIIT protocols producing the most robust—though not necessarily longest-lasting—improvements [2,7,8,13,15,19,24,34,41,45,46,59,60,63,72,73,84,91,94].

Third, acute changes in BDNF, lactate, catecholamines, blood pressure, and cerebral oxygenation appear to contribute to these cognitive enhancements, although no single mechanistic pathway fully explains the observed effects [1,2,3,5,6,7,8,9,10,17,18,21,22,23,25,28,30,39,41,50,52,56,57,58,59,64,69,70,73,79,80,86,87,88,89,93,96,97,98,99,100]. Rather, findings support a multi-mechanistic model in which acute exercise induces a transient state of neurobiological readiness—marked by increased neurotrophic signaling, improved cerebrovascular responsiveness, and greater prefrontal neural efficiency.

Fourth, cognitive benefits are observed across the lifespan, with older adults demonstrating improvements comparable to younger individuals, although with greater variability and potentially distinct mechanistic contributions [11,15,18,21,22,23,33,35,42,44,49,50,52,63,69,72,80,85,90,98]. Fitness level, environmental factors, hypoxia, and neurobiological responsiveness further shape the magnitude and durability of effects [22,28,33,37,43,56,57,61,69,71,73,75,76,94].

These findings highlight skeletal muscle as an active endocrine and metabolic organ, with acute contraction-induced signaling (e.g., myokines, lactate, catecholamines) representing a plausible mechanistic link between exercise and cognitive enhancement.

Overall, the available evidence positions acute exercise as a reliable, rapidly acting strategy to enhance cognitive performance, with promising implications for learning, occupational functioning, rehabilitation, and healthy aging.

Future research should prioritize standardized protocols, larger and preregistered mechanistic trials, multimodal imaging, and stratification by fitness, sex, age, and genetic or neurobiological profiles. Together, these steps will strengthen mechanistic insight and accelerate the translation of acute exercise into practical, personalized cognitive enhancement strategies.

## Figures and Tables

**Table 1:** Overview of study domains, populations, exercise modes and outcomes in included studies (N = 101) *.

Domain	Typical Population	Typical Exercise Modes	Typical Outcomes	Representative Studies (Examples)
Executive function/inhibitory control	Young healthy adults, students	Cycling, treadmill running, HIIT/HIIE, short moderate sessions Stroop, Flanker, Trail Making, task-switching	Majority show small–moderate acute improvement in RT with unchanged or slightly improved accuracy	Yanagisawa et al., 2010 (PFC activation + Stroop) [2] Kujach et al., 2018 (HIIT + executive + fNIRS) [4] Tsukamoto et al., 2016 (HIIT vs. moderate) [6] Chang et al., 2017 (ERP + general cognitive facilitation) [48]
Working memory/visuospatial memory	Young adults, some older adults and postmenopausal women	Cycling (continuous or interval), combined aerobic + resistance, HIIE + WBV, dual-task treadmill n-back, visuospatial WM tasks, mental rotation, digit span	Around half report clear WM benefits; others neutral, very few detrimental effects	Martínez-Díaz et al., 2020 (WM + BDNF + HIIT) [7] Piepmeier et al., 2020 (memory + BDNF isoforms) [11] Zheng et al., 2022 (WM + cortical activity) [24] Tsai et al., 2014 (visuospatial attention + BDNF) [39]
Learning/consolidation & hippocampal tasks	Young adults and older adults	Cycling or running before or after learning; some multi-week interventions with acute sessions measured Face–name learning, associative learning tasks, vocabulary learning	Generally positive effects when exercise is performed before or close to learning, especially in older adults	Griffin 2011 et al., (hippocampal function + BDNF) [10] Nilsson et al., 2020 (BDNF → learning link, older adults) [12] Perini et al., 2016 (exercise promotes learning) [45] Schmidt-Kassow et al., 2013 (exercise during encoding → vocabulary learning) [80]
Global cognition/dual-task function in older adults	Healthy older women, older mixed cohorts	Tai-Chi, multicomponent training, aquatic or land treadmill, combined strength + power programs MMSE, global composite scores, dual-task gait; slower, broader tests	Acute effects small; most benefits seen when acute response is embedded in a short training program	So et al., 2024 (CBF + cognition older adults) [29] Olivo et al., 2021 (RCT acute exercise + cognition + perfusion) [37] Imaizumi et al., 2025 (multicomponent + BDNF older women) [62] Kargaran et al., 2021 (dual-task + BFR elderly) [101]
Cerebrovascular/hemodynamic and neuroimaging studies	Young and older adults; some APOE4 carriers, athletes	Cycling or running in MRI or NIRS setups; sometimes concurrent cognitive tasks Measures of CBF (ASL-MRI), MCAv, NIRS over PFC, oxygenated/deoxygenated Hb	Consistent pattern: increased PFC oxygenation and/or CBF at moderate loads; plateau or decline at higher intensities	Giles et al., 2014 (PFC oxygenation) [23] Olivo et al., 2021 (CBF + cognition) [37] Lucas et al., 2012 (perfusion + executive function) [59] Thackray et al., 2023 (MRI + CBF) [66]

* Full list of individual studies, with details per trial, is provided in Supplementary Table S1. Abbreviations: APOE4—Apolipoprotein E ε4 allele; ASL-MRI—Arterial Spin Labeling Magnetic Resonance Imaging; CBF—Cerebral Blood Flow; Hb—Hemoglobin; HIIE—High-Intensity Interval Exercise; HIIT—High-Intensity Interval Training; MCAv—Middle Cerebral Artery Velocity; MMSE—Mini-Mental State Examination; MRI—Magnetic Resonance Imaging; NIRS—Near-Infrared Spectroscopy; PFC—Prefrontal Cortex; RT—Resistance Training; WBV—Whole-Body Vibration; WM—Working Memory.

**Table 2:** Cognitive domains relevant to acute and long-term exercise effects.

Cognitive Domain	Representative Tests	Likely Affected Time Horizon	Underlying Mechanisms/Notes
**Executive Function**	Stroop, Trail Making Test Part B, Wisconsin Card Sorting, Flanker	Acute + Long term	Increase in prefrontal activation and catecholamine release (acute); Increase in BDNF and functional connectivity (chronic)
**Attention/Processing Speed**	Trail Making Test Part A, Symbol Digit Modalities, Choice Reaction Time	Acute + Long term	Increased arousal, noradrenergic activity (acute); enhanced cerebrovascular flow and white matter integrity (chronic)
**Working Memory**	n-back, Digit Span backward, Letter-Number Sequencing	Acute ± Long term	Transient dopamine surge, PFC recruitment (acute); synaptic plasticity and hippocampal–prefrontal coupling (chronic)
**Episodic Memory**	Rey Auditory Verbal Learning, Word List Recall, Paired Associate Learning	Long term > Acute	Hippocampal neurogenesis, BDNF-mediated synaptic strengthening
**Global Cognition**	MMSE, MoCA, CDR-SB, CAMCOG	Long term	Integrative outcome—reflects cumulative effects on executive, memory, and speed domains
**Psychomotor Function**	Simple or choice reaction time, tapping tasks	Acute	Sensitive marker for arousal and fatigue; confounder control useful
**Language/Visuospatial Function**	Boston Naming, Clock Drawing, Rey–Osterrieth Figure	Long term	Less sensitive to exercise; useful for dementia subtype differentiation (AD vs. VaD)

Abbreviations: AD—Alzheimer’s Disease; BDNF—Brain-Derived Neurotrophic Factor; CAMCOG—Cambridge Cognitive Examination; CDR-SB—Clinical Dementia Rating—Sum of Boxes; MMSE—Mini-Mental State Examination; MoCA—Montreal Cognitive Assessment; PFC—Prefrontal Cortex; VaD—Vascular Dementia.

**Table 3:** Acute exercise prescriptions and cognitive effects (scoping summary).

Exercise Type	Intensity	Typical Paradigms	Main Cognitive Effects	Representative Examples
Moderate continuous aerobic exercise	20–30 min cycling or treadmill at ~50–70% VO_2_max or around anaerobic threshold	Stroop, Trail Making, Flanker, working-memory tasks during or shortly after exercise	Robust but modest improvements in RT on executive tasks; accuracy largely unchanged	Yanagisawa 2010 [2], Lucas 2012 [59], Córdova 2009 [1]
High-intensity interval/HIIT/HIIE	Short HIIE protocols (e.g., 8–10 × 30–60 s at 80–100% VO_2_peak with active rest)	Stroop, n-back, WCST, working memory and reaction time tasks post-exercise	Similar or slightly larger acute improvements in executive function; often more pronounced/longer-lasting during recovery; some evidence of “sweet spot” before fatigue.	Hwang 2016 [9], Tsukamoto 2016 [6], Martínez-Díaz 2020 [7], Slusher 2018 [5]
Resistance/power and combined (aerobic + resistance)	Single resistance sessions (3–5 sets of 8–12 reps) or combined RE + continuous/interval aerobic	Executive function and WM (Flanker, n-back, Stroop)	Mixed but generally positive: several report faster RT and improved WM; some show speed–accuracy trade-off at very high intensity.	Baumgartner 2025 [35], Coelho-Júnior 2020 [100]
Mind–body/multicomponent/dual-task programs	Tai-Chi, multicomponent circuit + Pilates, dual-task training with treadmill walking ± BFR	Global cognition, dual-task gait, QoL, MMSE, executive composites	Acute session often part of short program; acute improvements modest, but repeated exposure linked to better dual-task performance and QoL; BDNF sometimes increases across program.	Morawin 2021 [51], Imaizumi 2025 [62]
Hypoxia/special conditions (aquatic treadmill, eccentric cycling, balance tasks, open-skill games)	Exercise in hypoxia, aquatic treadmill vs. land, eccentric vs. concentric cycling, badminton vs. running	Executive tasks, attention, reaction time; some pure physiological paradigms	Results mixed: hypoxia often slows RT despite increased BDNF; aquatic treadmill and eccentric cycling show similar or slightly better cognitive benefits at lower HR; open-skill (e.g., badminton) seems to boost inhibitory control more than closed-skill running at matched intensity.	Lefferts 2016 [60], Piotrowicz [28], Borot 2024 [31], Takahashi 2023 [70]

Abbreviations: BDNF—Brain-Derived Neurotrophic Factor; BFR—Blood Flow Restriction; HIIT/HIIE—High-Intensity Interval Training/High-Intensity Interval Exercise; MMSE—Mini-Mental State Examination; QoL—Quality of Life; RE—Resistance Exercise; RT—Reaction Time; VO_2_max—Maximal Oxygen Uptake; WCST—Wisconsin Card Sorting Test; WM—Working Memory Influence of exercise intensity.

**Table 4:** Cognitive domains and tests used across acute exercise studies.

Cognitive Domain	Typical Task(s)	Number of Studies Using This Approach (Qualitative)	Overall Pattern
Executive function/inhibitory control	Color–word Stroop, CWST, Flanker, WCST, Stroop-like Stroop interference tasks	Most central domain; many studies	Acute moderate and high-intensity exercise generally improves RT (smaller interference cost) with little change in accuracy; effects may persist 20–30 min post-exercise in HIIE/HIT designs.
Working memory	n-back (visuospatial and verbal), digit span, visuospatial WM tasks, delayed matching	Frequently studied, especially in college students and older adults	Many show improved WM immediately after exercise; particularly when exercise intensity is moderate–vigorous; some null findings when tasks are very easy or very hard.
Learning and consolidation	Face–name matching, vocabulary learning, visual discrimination learning, thumb-abduction motor learning	Fewer studies but central to “acute priming” hypothesis	Exercise before or in close temporal proximity to learning sessions often facilitates acquisition and/or consolidation, especially when training is repeated over days
Attention/vigilance/psychomotor speed	Simple and choice RT, sustained attention to response, visuomotor tracking	Common in eccentric vs. concentric, workplace-style prolonged sitting vs. breaks	Simple RT and vigilance usually improve or are maintained with intermittent exercise; prolonged sitting alone leads to deterioration.
Global cognitive function/composite scores	MMSE, MoCA, global composites, dual-task walking scores	Primarily in older women/older adults over short programs	Acute changes small; most signal comes from repeated sessions (training effects), but acute BDNF/cytokine or hemodynamic responses may predict responders.

Abbreviations: BDNF—Brain-Derived Neurotrophic Factor; CWST—Color–Word Stroop Test; HIIE—High-Intensity Interval Exercise; HIT—High-Intensity Training; MMSE—Mini-Mental State Examination; MoCA—Montreal Cognitive Assessment; RT—Reaction Time; WCST—Wisconsin Card Sorting Test.

**Table 5:** Brain hemodynamics and imaging outcomes in acute exercise studies.

Technique	Example Studies	Main Acute Pattern
Transcranial Doppler (MCAv, ICA blood flow)	Lucas 2012 [59], Shoemaker 2020 [25], Horiuchi 2023 [61]	Moderate exercise increases MCAv/ICA flow; prolonged sitting decreases CBF and worsens executive function; intermittent squats or bouts of exercise blunt this decline
Near-infrared spectroscopy (NIRS/fNIRS over PFC and parietal areas)	Yanagisawa 2010 [2], Giles 2014 [23], Zheng 2022 [24], Damrongthai 2021 [21], Lefferts 2016 [60], Doneddu 2024 [67]	Mild–moderate intensities: increased oxygenated Hb in PFC and sometimes parietal regions, paralleling better Stroop/WM performance Very high intensity or hypoxia: PFC oxygenation may decline and cognitive performance plateaus or worsens
MRI (ASL perfusion, BOLD task fMRI, structural measures)	Nilsson 2020 [12], Olivo 2021 [37], Mast 2022 [58], Thackray 2023 [66], Vidoni 2022 [53]	Gray matter CBF sometimes decreases shortly after exercise but with regional increases (e.g., hippocampus, motor cortex) Exercise can alter hippocampal/medial temporal perfusion and modulate food-cue reactivity; however, acute perfusion changes and cognition are not always tightly coupled
EEG/ERPs (P3, N2, CNV, etc.)	Chang 2017 [48], Tsai 2016, Tsai 2025 [3,46], Li 2024 [33], Tsai 2014 [39]	Acute exercise often increases P3 amplitude and/or shortens P3 latency in executive/WM tasks, consistent with more efficient resource allocation CNV changes point to improved preparatory attention, especially in higher-fit participants.
Combined hemodynamic–behavioral paradigms	Multiple Stroop + fNIRS studies, eccentric vs. concentric cycling, open- vs. closed-skill sports (e.g., badminton vs. running)	Most show that conditions which improve executive function also either increase task-related PFC activation or allow similar performance with lower activation (i.e., neural efficiency), depending on paradigm

Abbreviations: BOLD—Blood Oxygen Level-Dependent (MRI signal); CBF—Cerebral Blood Flow; CNV—Contingent Negative Variation (ERP component); EEG—Electroencephalography; ERP—Event-Related Potential; fMRI—Functional Magnetic Resonance Imaging; fNIRS—Functional Near-Infrared Spectroscopy; Hb—Hemoglobin; ICA—Internal Carotid Artery; MCAv—Middle Cerebral Artery Velocity; MRI—Magnetic Resonance Imaging; N2—N2 ERP component; NIRS—Near-Infrared Spectroscopy; P3—P3 (P300) ERP component; PFC—Prefrontal Cortex; WM—Working Memory.

**Table 6:** Molecular markers assessed in acute exercise–cognition studies (from abstracts).

Marker/Pathway	Studies Mentioning Marker (Examples)	Direction of Acute Response	Relation to Cognitive Outcomes (as Reported)
BDNF (serum/plasma, sometimes proBDNF vs. mBDNF)	Piepmeier 2020 [11], Hwang 2016 [9], Baumgartner 2024 [32], Morris 2024 [49]	Typically increases acutely after moderate–vigorous aerobic or HIIT; sometimes intensity- or fitness-dependent; some protocols show no change.	Correlations with cognitive change are inconsistent: a few studies report associations (e.g., better learning when BDNF rises pre-training, or links with task-switching), while others find no direct correlation
Other neurotrophic/anabolic factors (IGF-1, VEGF, CTSB, GPLD1, Klotho, growth hormone)	Tsai 2016 (IGF-1, HGH) [3], Bekkos 2025 (Klotho, GPLD1) [44], Vidoni 2022 (IGF-1, VEGF) [53], Imaizumi 2025 (BDNF in training programs) [62]	Often rise acutely after exercise, especially with higher intensity or combined modes.	Evidence linking these changes with acute cognitive effects is preliminary; mostly exploratory correlations.
Cytokines and inflammatory markers (IL-6, IL-1ra, CRP, TNFα, TNFRII, CAF, P3NP, etc.)	Kuhne 2023 (IL-6, IL-1ra, IL-4, IFN-γ) [19], Morawin 2021 (CRP, TNFα, TNFRII) [51]	Acute exercise produces transient inflammatory changes; in some studies, greater IL-6/IL-1ra responses correlate with better within-session learning	Evidence suggesting an immune contribution to plasticity.
Metabolic/stress markers (lactate, cortisol, catecholamines)	Lefferts 2016 (DHEA, NSE) [60], Baumgartner 2024 (lactate) [32], Piotrowicz 2020 (catecholamines) [28], Coco 2016 (lactate) [99]	Lactate frequently rises strongly with HIIT/HIIE and high-intensity resistance, and in some studies statistically mediates RT improvements; others show speed–accuracy trade-offs or negative effects at very high loads.	Cortisol often increases and may accompany better working memory in some HIIT protocols, but high CO_2_/cortisol in hypercapnia or severe hypoxia tends to impair performance.
Bone/muscle—OC, ucOC, cOC, myokines	Tsai 2025 (osteocalcin isoforms, irisin) [46], Morawin 2021 [51], multicomponent and Tai-Chi trials	Single bouts influence osteocalcin, irisin and related bone–muscle markers	Association with acute cognitive change is exploratory and mostly non-significant so far

Abbreviations: BDNF—Brain-Derived Neurotrophic Factor; CAF—Cathepsin A Fragment (or Caffeine Depending on Context—here it is Cathepsin A Fragment, as used in exercise–biomarker studies); cOC—Carboxylated Osteocalcin; CRP—C-Reactive Protein; CTSB—Cathepsin B; DHEA—Dehydroepiandrosterone; HIIE—High-Intensity Interval Exercise; HIIT—High-Intensity Interval Training; IGF-1—Insulin-Like Growth Factor 1; IL-1ra—Interleukin-1 Receptor Antagonist; IL-6—Interleukin-6; mBDNF—Mature Brain-Derived Neurotrophic Factor; NSE—Neuron-Specific Enolase; OC—Osteocalcin (total); P3NP—Procollagen Type III N-Terminal Peptide; proBDNF—Pro-Brain-Derived Neurotrophic Factor; RT—Resistance Training; TNFα—Tumor Necrosis Factor Alpha; TNFRII—Tumor Necrosis Factor Receptor II; ucOC—Undercarboxylated Osteocalcin; VEGF—Vascular Endothelial Growth Factor.

**Table 7:** Conceptual summary of acute exercise effects on cognition and mechanistic markers.

Key Theme	Summary
“Goldilocks” intensity window	Across studies, moderate to moderately high intensities (around the anaerobic threshold to classic HIIT/HIIE) most consistently improve executive function and working memory. Too light = small/no effect; too hard/hypoxic = risk of slower RT or accuracy decrements.
Timing vs. learning	When exercise is performed immediately before or interleaved with cognitive training, some studies show better learning and consolidation, especially in older adults. Order effects (exercise before vs. after) may matter via BDNF and other neuromodulators.
Fitness as a moderator	Higher cardiorespiratory fitness often amplifies cognitive and neuroelectric gains (e.g., larger P3 changes, smaller switching costs) and may shape the BDNF response.
BDNF is important but not sufficient	BDNF nearly always moves in the “right” direction with vigorous aerobic sessions, but cognitive benefits frequently occur without strong BDNF–behavior correlations. Other mediators (lactate, catecholamines, immune markers, cortisol) likely contribute.
Brain hemodynamics: PFC and hippocampus	Moderate exercise improves PFC oxygenation and may transiently alter hippocampal perfusion. These changes align with improved executive performance in many, but not all, paradigms At very high intensity or in hypoxia PFC oxygenation can fall and performance suffers.
Older vs. younger adults	Older adults generally benefit in similar domains (executive function, WM), but effects are smaller and sometimes only evident when acute sessions are embedded in multi-week programs. BDNF and cytokine responses may predict “responders”.
Task dependency	Executive and WM tasks with some difficulty (Stroop, Trail Making, n-back) are more sensitive to acute exercise than very easy or very hard tasks, which often show ceiling or floor effects.

Abbreviations: BDNF—Brain-Derived Neurotrophic Factor; HIIE—High-Intensity Interval Exercise; HIIT—High-Intensity Interval Training; P3—P300 Event-Related Potential Component; PFC—Prefrontal Cortex; RT—Resistance Training.

## Data Availability

The original contributions presented in this study are included in the article/Supplementary Material. Further inquiries can be directed to the corresponding author.

## Supplementary Materials

The following supporting information can be downloaded at: https://www.mdpi.com/article/10.3390/muscles5020025/s1, Table S1: Acute Exercise Effects_Titles and Abstracts_selected.xlsx.

## Abbreviations

The following abbreviations are used in this manuscript:

AD	Alzheimer’s Disease
APOE4	Apolipoprotein E ε4 Allele
ASL	Arterial Spin Labeling
ASL-MRI	Arterial Spin Labeling Magnetic Resonance Imaging
BDNF	Brain-Derived Neurotrophic Factor
BFR	Blood Flow Restriction
BOLD	Blood Oxygenation Level-Dependent (fMRI signal)
CAF	Cathepsin A Fragment
CAMCOG	Cambridge Cognitive Examination
CBF	Cerebral Blood Flow
CDR-SB	Clinical Dementia Rating—Sum of Boxes
cOC	Carboxylated Osteocalcin
CO_2_	Carbon Dioxide
CNV	Contingent Negative Variation (ERP component)
CRP	C-reactive Protein
CTSB	Cathepsin B
CWST	Color–Word Stroop Test
DHEA	Dehydroepiandrosterone
EEG	Electroencephalography
ERP	Event-Related Potential
fMRI	Functional Magnetic Resonance Imaging
fNIRS	Functional Near-Infrared Spectroscopy
GABA	Gamma-Aminobutyric Acid
GPLD1	Glycosylphosphatidylinositol-Specific Phospholipase D1
Hb	Hemoglobin
HbO/HbR	Oxygenated/Deoxygenated Hemoglobin
HIIE	High-Intensity Interval Exercise
HIIT	High-Intensity Interval Training
HIT	High-Intensity Training
ICA	Internal Carotid Artery
IFN-γ	Interferon-gamma
IGF-1	Insulin-Like Growth Factor 1
IL-1ra	Interleukin-1 Receptor Antagonist
IL-4	Interleukin-4
IL-6	Interleukin-6
MCA	Middle Cerebral Artery
MCAv	Middle Cerebral Artery Velocity
MCI	Mild Cognitive Impairment
mBDNF	Mature Brain-Derived Neurotrophic Factor
MMSE	Mini-Mental State Examination
MoCA	Montreal Cognitive Assessment
MRI	Magnetic Resonance Imaging
N2	N2 ERP Component
NIRS	Near-Infrared Spectroscopy
NSE	Neuron-Specific Enolase
OC	Osteocalcin (total)
P3/P300	P3 (P300) ERP Component
P3NP	Procollagen Type III N-Terminal Peptide
PFC	Prefrontal Cortex
PRISMA-ScR	Preferred Reporting Items for Systematic Reviews and Meta-Analyses—Scoping Review
QoL	Quality of Life
RE	Resistance Exercise
RT	Resistance Training
SART	Sustained Attention to Response Task
SMA	Supplementary Motor Area
T1D	Type 1 Diabetes
TCD	Transcranial Doppler
TMT	Trail Making Test
TNFα	Tumor Necrosis Factor Alpha
TNFRII	Tumor Necrosis Factor Receptor II
ucOC	Undercarboxylated Osteocalcin
VaD	Vascular Dementia
VEGF	Vascular Endothelial Growth Factor
VO_2_max/VO_2_peak	Maximal/Peak Oxygen Uptake
WBV	Whole-Body Vibration
WCST	Wisconsin Card Sorting Test
WM	Working Memory
